# The relationship between DXA-based and anthropometric measures of visceral fat and morbidity in women

**DOI:** 10.1186/1471-2261-13-25

**Published:** 2013-04-03

**Authors:** Kenan Direk, Marina Cecelja, William Astle, Phil Chowienczyk, Tim D Spector, Mario Falchi, Toby Andrew

**Affiliations:** 1 Department of Twin Research and Genetic Epidemiology, London, UK; 2 Department of Clinical Pharmacology, King’s College London, School of Medicine, London, UK; 3 Epidemiology, Biostatistics and Occupational Health, McGill University, Montreal, Canada; 4 Department of Genomics of Common Disease, School of Public Health, Imperial College London, Hammersmith Hospital, London, UK

**Keywords:** Visceral fat, Adiposity, DXA, Type 2 diabetes, Hypertension, Subclinical atherosclerosis, Liver function

## Abstract

**Background:**

Excess accumulation of visceral fat is a prominent risk factor for cardiovascular and metabolic morbidity. While computed tomography (CT) is the gold standard to measure visceral adiposity, this is often not possible for large studies - thus valid, but less expensive and intrusive proxy measures of visceral fat are required such as dual-energy X-ray absorptiometry (DXA). Study aims were to a) identify a valid DXA-based measure of visceral adipose tissue (VAT), b) estimate VAT heritability and c) assess visceral fat association with morbidity in relation to body fat distribution.

**Methods:**

A validation sample of 54 females measured for detailed body fat composition - assessed using CT, DXA and anthropometry – was used to evaluate previously published predictive models of CT-measured visceral fat. Based upon a validated model, we realised an out-of-sample estimate of abdominal VAT area for a study sample of 3457 female volunteer twins and estimated VAT area heritability using a classical twin study design. Regression and residuals analyses were used to assess the relationship between adiposity and morbidity.

**Results:**

Published models applied to the validation sample explained >80% of the variance in CT-measured visceral fat. While CT visceral fat was best estimated using a linear regression for waist circumference, CT body cavity area and total abdominal fat (*R*^2^ = 0.91), anthropometric measures alone predicted VAT almost equally well (CT body cavity area and waist circumference, *R*^2^ = 0.86). Narrow sense VAT area heritability for the study sample was estimated to be 58% (95% CI: 51-66%) with a shared familial component of 24% (17-30%). VAT area is strongly associated with type 2 diabetes (T2D), hypertension (HT), subclinical atherosclerosis and liver function tests. In particular, VAT area is associated with T2D, HT and liver function (alanine transaminase) independent of DXA total abdominal fat and body mass index (BMI).

**Conclusions:**

DXA and anthropometric measures can be utilised to derive estimates of visceral fat as a reliable alternative to CT. Visceral fat is heritable and appears to mediate the association between body adiposity and morbidity. This observation is consistent with hypotheses that suggest excess visceral adiposity is causally related to cardiovascular and metabolic disease.

## Background

Body fat distribution, particularly abdominal adiposity, is strongly associated with a range of chronic metabolic and cardiovascular morbidities. The abdomen includes depots of both subcutaneous and visceral adipose tissue, which are distinct tissues that present different gene expression [1], endocrinological profiles [2] and pathogenicity [3]. The detrimental effect of excess fat accumulation is contingent on body fat distribution. Kim *et al.*[3] have shown that the diabetic phenotype of obese leptin knock out mice can be rescued by shifting fat storage away from the viscera and into the subcutaneous depot. The high ratio of subcutaneous to visceral fat in this mouse model is in contrast to the familial partial forms of lipodystrophy observed in humans, a pathology characterised by a redistribution of adipose tissue to the intra-abdominal region, giving rise to severe metabolic abnormalities as a consequence. These extreme cases illustrate the potent effect that adipose distribution can have upon health [4,5]. The accumulation of visceral fat may also play a role in the aetiology of cardiovascular and metabolic disease.

Adiposity can be measured with a variety of techniques such as dual-energy X ray absorptiometry (DXA), computed tomography (CT) and magnetic resonance imaging (MRI). In whole body composition analysis, CT is regarded as the gold standard and as such has been used extensively as a benchmark for the quantitative assessment of subcutaneous and visceral adiposity. However, CT and MRI are limited in large research studies due to cost [6] and radiation exposure [7]. As such, there is a continued effort to identify informative adiposity measures that are safe, easily acquired and show robust correlation with CT-measured visceral fat content [8-11].

Family and twin studies can be used to estimate the heritability (the proportion of trait variance explained by genetic factors) and the relative importance of genetic and shared environmental factors in influencing phenotypic variance, by contrasting trait covariance between relatives with degree of relatedness. Establishing the heritable basis of a trait is also an important consideration prior to gene mapping studies [12].

The aims of this study are to 1) identify and estimate a valid DXA and anthropometric based measures of visceral fat that can be used in this and future epidemiological studies, 2) estimate the heritability and the degree of shared familial environment that influences visceral fat accumulation and 3) to assess whether association between body composition measures of adiposity and morbidities can be fully explained by visceral fat. We hypothesise that if visceral fat is causally related to morbidity, we would expect visceral fat to remain associated with morbidity conditional upon all other measures of body fat distribution.

## Methods

### Subjects and data collection

St. Thomas’ Research Ethics Committee approved the study (EC96/439 Twins UK) and all participants provided informed written consent. Participants were female volunteers enrolled in the TwinsUK resource, originally established in 1992 to study the heritability of osteoarthritis and osteoporosis in women, but subsequently healthy male and female twins have been recruited (mainly via national advertisements) without respect to any particular disease [13]. While the twins are largely representative of the age-matched UK female population [14], our findings relating to adiposity are restricted to Caucasian women over 40 years old with a BMI ≤ 35.

The validation sample comprised 54 female individuals over the age of 40 years with both CT and DXA data, but was otherwise unselected. Previously reported linear regression models [10,11,15] and adiposity indices [8] were assessed for this sample to identify the most predictive combination of DXA abdominal fat and anthropometry measures required to estimate visceral fat. A validated best-fit model was then used to realise an estimate of abdominal visceral fat for a study sample of 3,457 female individuals (533 monozygotic twin pairs, 1102 dizygotic pairs, 187 singletons), where date-matched DXA abdominal fat and anthropometric data were available.

### CT

Subjects were placed in the supine position and a truncal CT scan was performed using a CT helical scanner (Brilliance CT component of the Philips Precedence SPECT/CT system, Philips, Eindhoven, Netherlands) with slice thickness set at 5 mm. The total cross sectional area (CSA) was intersected at the level of the intervertebral disc between lumbar vertebrae four and five (L4:L5). CT scan were analysed using Osirix X imaging software, version 3.7.1 (Pixmeo, Switzerland) to provide high-resolution 3-dimensional images. The body cavity CSA was obtained by tracing the outer contour of the abdominal wall and adipose tissue within this area was identified as having an attenuation value between −190 and −30 Hounsfield units [16]. For the 54 subjects with CT and DXA scan data (“validation sample” from here on in), visceral fat was directly measured as a pixel count using CT single slice area (VAT) (area). VAT area was defined as (visceral pixel count)/(total body cavity pixel count) × BC CSA. Single slice visceral fat area and volume are highly correlated [17,18].

### DXA

Baseline whole body DXA scans were performed for 3,457 female twins aged 40–80 years (“study sample” from here on in), in the supine position, using a fan beam X-ray bone densitometer (QDR-4500 W, Hologic, Massachusetts) and analysed with QDR System Software Version 12.6 (Hologic, Inc., Massachusetts). The DXA scanner was calibrated daily with a spine phantom and weekly using the step phantom as instructed by the manufacturer’s guidelines. The region of interest for body composition analysis was manually defined in a similar manner to Bertin *et al.*[8]. The abdominal region was delineated by an upper horizontal border located at half of the distance between acromions and external end of iliac crests and a lower border determined by the external end of iliac crests, but the lateral borders were delineated by the inner body wall rather than laterally past any trunk soft tissue. DXA fat mass from this abdominal region was recorded and abdominal transverse internal and external diameters were measured as defined by Bertin *et al.*[8].

### Anthropometry

Anthropometrical measurements for both the validation and study samples were height, weight, BMI and waist circumference [14]. Waist circumference for the validation sample was based upon the transverse circumference of CT body scan image at the waist, while the study sample was a tape measurement taken at the same time as the DXA scan. CT images were used to measure for sagittal depth, body cavity, subcutaneous fat and total abdominal fat cross sectional areas, transverse internal and external diameters (TID and TED respectively) and subcutaneous fat width (SFW). Subcutaneous fat width (SFW) was estimated as SFW = (TED – TID)/2, which was also used as an estimate for skin fold as a calliper skin fold measure was not taken for the TwinsUK sample. The 54 subjects were also measured for TED and TID (with SFW derived) using DXA software to draw a horizontal line on the DXA image at the upper most point of the iliac crest, which was converted from pixel to mm length by multiplying by a constant of 2.048 (DXA software support, Vertec Scientific Limited).

### Type 2 diabetes

Subjects were identified as type 2 diabetic (T2D) if they were classified as hyperglycaemic for one or more of the following diagnostics: fasting glucose serum concentration > 7 mmol/L, fasting 2 hour oral glucose tolerance test > 11.1 mmol/L or glycosylated haemoglobin > 48 mmol/mol. Questionnaire data on physician diagnosis and medication was also used to identify self-reported cases of T2D (2010–2011). Fasting plasma glucose was measured by enzymatic colorimetric slide assay (Johnson and Johnson Clinical Diagnostic Systems, Amersham UK).

### Hypertension

Blood pressure was measured twice using an automatic blood pressure monitor (Omron Healthcare, Inc., Bannockburn, IL) using data from the same visit as the DXA scan. Individuals were classified as having hypertension (HT), if currently receiving anti-hypertensive medication and/or the repeated systolic/diastolic blood pressure was greater than 140/90 mm Hg.

### Carotid intima-media thickness

Carotid intima-media thickness (cIMT) was measured as previously described by Cecelja *et al.*[19] and the quantitative trait was dichotomised at the 75th percentile as a surrogate for subclinical atherosclerosis [20,21]. In brief, the left and right carotid and femoral arteries were visualized with B-mode ultrasound (Siemens CV70, Siemens, Erlangen, Germany, with 13-MHz vascular probe). Common carotid IMT was measured in the near and far walls, 1 to 2 cm proximal to the carotid bifurcation with automated wall-tracking software (Medical Imaging Applications, Coralville, Iowa) during diastole in an area free of overt plaque. Mean values of IMT in the near and far walls of both arteries were used for analysis.

### Liver function tests

Data on liver function test (LFT) proteins were available for 87% of the study sample (for details, see Rahmioglu *et al.*[22]). Abnormal liver function as assessed by LFTs can be used to assist diagnosis of non-alcoholic fatty liver disease (NAFLD) [23]. Serum concentrations of alanine transaminase (ALT), alkaline phosphatase (ALK), total bilirubin (BIL) and γ-glutamyl transpeptidase (GGT) were used in this study as markers of liver function. Of the four tests, ALT is the most sensitive marker for liver cell damage (as a consequence of disease or drug use), while ALK and GGT are more indicative of cholestatic injury and BIL, haem catabolism [24], [25]. The upper limit of normal threshold used for each protein were: ALT: 39.4 IU/L [26], ALK: 81 IU/L (taken as the 75% percentile of the sample distribution), BIL: 17.1 μmol/L [27], GGT: 33 IU/L [28].

### Statistical analyses

#### Validation sample

Multiple regression analyses [10,11,15] and indices of adiposity were assessed [8] to address two questions relating to the estimation of total abdominal visceral fat using DXA adiposity and a range of anthropometric measures: 1) which of these predictive models for the “gold standard” CT measure of visceral fat, best fit our validation sample of 54 females; and 2) in relation to previous discussions [8], can visceral fat be reliably estimated based upon anthropometry alone. CT and DXA scans for the same individuals were date-matched to between 0.23 - 2.3 years of one another. The difference in scan date for the validation sample was included in all visceral fat regression models as a nuisance factor. A Bland-Altman analysis was conducted to assess if the predicted VAT error term was constant or varied across the range of CT-measured VAT area.

#### Heritability analysis (study sample)

The classical twin model was used to estimate the relative influence of genetic and environmental factors upon individual variation about the visceral fat sample mean [29]. Using variance components analysis, the total phenotypic variance was partitioned into estimates of the additive polygenic (A), dominant (D) genetic and shared familial environment (common; C) and unique to the individual, environmental and measurement error (E) components using genetically informative data. The model assumes no epistasis, gene-environment correlation or interactions and that shared environmental factors are not confounded by zygosity (the equal environments assumption). Provided these assumptions hold, on the basis that MZ twins share identical genes and DZ twins share, on average, half their segregating genes, twin data can be used to infer heritability and shared familial estimates. Maximum likelihood models were implemented using Mx [30].

In addition to the univariate heritability analysis, we also conducted a bivariate variance components analysis between the realised estimate of VAT area and total abdominal fat as an indirect means to further validate the proxy measure of VAT. Bivariate analysis facilitates a test of genetic correlation between two variables, but also whether each variable has a specific genetic component that is not shared by the two traits. We used the specific test to assess if there was evidence for a genetic component that is unique to VAT area and not shared with DXA total abdominal fat [30].

#### Morbidity association with adiposity (study sample)

Type 2 diabetes, hypertension and liver function were associated with the different measures of adiposity variables and age using logistic regression using a cross-sectional study design. By contrast, prospective incident subclinical atherosclerosis (cIMT) was modelled using Cox regression and baseline measures of visceral fat taken on average 10 years (range 5–16 years) previously. Since there was no baseline examination of cIMT, we could not exclude the small proportion of individuals who may have already developed subclinical atherosclerosis at baseline. To the extent this was true, the study design for these individuals would have been also cross-sectional rather prospective.

LFTs were performed using blood samples collected at the same visit as the DXA scan. The mean, standard deviation and median were all examined to identify potential batch effects across years, but no obvious trend was identified. Therefore the year of visit was categorised as quintiles and included in all analyses as a categorical confounding variable.

Co-linearity between the explanatory measures of VAT area, DXA total abdominal fat, BMI and age was assessed using pairwise correlations and residuals analysis [31,32]. A variance inflation factor (VIF) was calculated for these data, with a score of ≥ 10 sometimes used as a (conservative) threshold indicator for potentially problematic co-linearity between model explanatory variables [32]. To assess consistency of results, the multiple regression analyses were repeated using random subgroups of data and using quantitative traits for all subjects, where morbidity had been defined using a quantitative trait threshold (i.e. systolic and diastolic blood pressure and LFTs).

Parsimonious best-fit multiple regression models for morbidity were identified by a) using a likelihood ratio test (LRT) to assess the contribution of each explanatory variable to the full model; b) assessing model fit using pseudo-*R*^2^ for T2D, HT and LFTs and the Wald statistic for incident cIMT, and c) the implementation of a linear residuals analyses [31] to assess whether adiposity measures are independent of morbidity, when conditioned upon on visceral fat. For example, to assess if VAT completely mediates the association between BMI and T2D, secondary residuals analysis involved taking the residuals for T2D on the logit scale and the ordinary least squares residuals for BMI, both with respect to VAT area and age. Ordinary least squares (OLS) residuals for DXA total abdominal fat were also taken with respect to VAT area and age. The secondary residuals analysis was implemented using linear regression of T2D residuals upon BMI and DXA residuals. No evidence of association between residuals would imply that BMI/DXA association with morbidity is primarily mediated via VAT area, while a significant residual association demonstrates either that BMI/DXA are associated with morbidity through a path not mediated by VAT area, or that the parametric assumptions of the association models are false.

To facilitate assessment of the relative importance of adiposity measures associated with morbidity, unadjusted and adjusted odd ratios (OR) results are presented for VAT area (cm^2^), DXA total abdominal fat (kg) and BMI (kg/m^2^). To facilitate comparison between the different adiposity units, all adiposities were standardised for the regression analyses. Robust standard errors were estimated by grouping twin pairs using the cluster option in Stata to account for intra-family relatedness. All statistical analyses were performed using Stata version 11.1 (StatCorp, Texas).

## Results

### Estimation of visceral fat (validation sample)

Descriptive statistics for anthropometric and body composition for the validation and study samples are presented in Table 1. All subjects were female and over 40 years old at examination. The mean height, weight, BMI, DXA total abdominal fat, blood pressure, cIMT, liver function tests and visceral fat do not statistically differ between the two samples, although the mean age for the validation sample was six years older (p = 6.5 × 10^-11^) with a larger waist circumference (p = 2.05 × 10^-6^) than the study sample. The study sample prevalence and 95% confidence interval for morbidity related to the quantitative traits presented in Table 1 were as follows: T2D 0.046 (0.04-0.05), HT 0.08 (0.07-0.09), cIMT 0.27 (0.24-0.30), ALT 0.22 (0.21-0.24), ALK 0.27 (0.26-0.29), BIL 0.02 (0.02-0.03) and GGT 0.20 (0.18-0.21).

**Table 1:** Validation and study sample characteristics

	**Validation sample (n = 54)**				**Study sample (n = 3457)**			
	**Mean**	**SD**	**Min.**	**Max.**	**Mean**	**SD**	**Min.**	**Max.**
Age (years)	60.4	6.1	49.3	72.8	54.2	8.3	40.0	79.5
Weight (kg)	65.7	9.4	48.4	87.4	66.6	11.8	35.6	139.5
Height (m)	1.62	0.06	1.48	1.75	1.62	0.06	1.39	1.82
Waist circumference (cm)	88.0	9.9	66.6	111.2	81.0	11.0	55.0	134.0
Sagittal depth (cm)	21.8	3.2	15.9	31.1	-	-	-	-
Scan difference (years)	1.3	0.8	0.2	2.5	-	-	-	-
BMI (kg/m^2^)	25.1	3.8	19.2	33.8	25.6	4.5	15.1	51.7
Total abdominal fat (kg)	1.40	0.62	0.24	3.08	1.44	0.61	0.14	3.94
VAT area (cm^2^)	127.8	52.1	37.7	279.5	144.6	49.6	37.7	347.4
Diastolic BP (mmHG)	77.3	8.3	61.0	95.5	75.9	8.9	47.5	108.0
Systolic BP (mmHG)	122.0	11.5	92.0	151.0	123.1	14.7	86.5	189.0
cIMT	0.68	0.08	0.53	0.82	0.67	0.11	0.30	1.11
ALT	23.9	11.8	3.0	68.0	26.7	11.4	2.5	217.3
ALK	64.8	19.0	26.0	114.0	71.2	18.2	23.5	218.9
BIL	9.5	3.7	5.7	23.5	8.7	3.0	1.0	30.5
GGT	30.3	17.4	12.0	65.3	27.9	21.7	3.0	359.0

Age, weight, height, body fat distribution and intermediate quantitative traits used to define clinical morbidity. Waist circumference for the validation sample is based upon the transverse circumference of CT body scan image at the waist, while the study sample is a tape measurement taken at the same time as the DXA scan. Abbreviations: *ALT*, alanine transaminase, *ALK*, alkaline phosphatase, *BIL*, bilirubin, *BP*, blood pressure, *cIMT*, carotid intima-media thickness, *GGT*, gamma-glutamyl transpeptidase, *SD*, standard deviation (between family).

The Pearson product moment correlation coefficients (*r*) between the different adiposity measures and CT measured VAT area for the validation sample are presented in Table 2. CT-VAT area was most strongly correlated with CT-measured body cavity cross sectional area (*r* = 0.85), sagittal depth (*r* = 0.84) and tape-measured waist circumference (*r* = 0.86) and DXA total abdominal fat (*r* = 0.79). Consistent with these data, we observed that reported models of visceral fat in the literature, whether linear regressions or derived anthropometric indices, all attempt to capture information about the body cavity volume (or area) in relation to the subcutaneous volume [8,10,11,15]. We used this insight to guide our choice of linear regression to estimate CT-measured visceral fat.

**Table 2:** Validation sample (n = 54) correlation coefficients between CT visceral adipose fat (VAT) area, anthropometric and abdominal fat measures

	**VAT**	**BC**	**Sub.CSA**	**Total CSA**	**SD**	**WC**	**TID**	**TED**	**SFW**	**DXA**	**BMI**
BC	**0.85**										
Sub. CSA	0.58	0.44									
Total CSA	0.81	0.80	0.90								
SD	**0.84**	0.80	0.84	0.96							
WC	**0.86**	0.77	0.83	0.94	0.94						
TID	0.66	0.72	0.46	0.67	0.61	0.65					
TED	0.66	0.56	0.89	0.87	0.82	0.85	0.62				
SFW	0.43	0.26	0.83	0.69	0.66	0.67	0.17	0.88			
DXA	**0.79**	0.56	0.68	0.74	0.76	0.77	0.56	0.75	0.6		
BMI	0.71	0.60	0.80	0.84	0.83	0.82	0.57	0.85	0.72	0.79	
Weight	0.67	0.64	0.77	0.84	0.76	0.81	0.69	0.88	0.68	0.69	0.86

All measures presented here are based upon CT scan images apart from DXA total abdominal fat, BMI and weight. Abbreviations and units: *BC*, body cavity area (cm^2^), *BMI*, body mass index (kg/m^2^), *CSA*, body cross-sectional area (cm^2^) at L4:L5, *DXA*, DXA-measured total abdominal fat (kg), *SD*, sagittal depth (cm), Sub.*CSA*, subcutaneous cross sectional area, *SFW*, subcutaneous fat width (cm), *TED*, transverse external diameter (cm), *TID*, transverse internal diameter (cm), *VAT*, visceral adipose tissue (cm^2^), *WC*, waist circumference (cm) derived from the CT-image, weight (kg).

Although the DXA scans were collected between 0.23 – 2.3 years after the CT scans for the validation sample, no evidence was observed for significant change in weight in these individuals nor was change in weight correlated with time lapse between scan dates (data not shown).

Table 3 presents the results for three previously published DXA-based regression models and anthropometric indices for estimating visceral fat, applied to the TwinsUK CT validation sample. The best-replicated regression models included DXA trunk fat and sagittal depth (*R*^2^ ≈ 0.8), while a combination of DXA and skin fold was less predictive of visceral fat. The best individual indices were functions of sagittal depth (SD), SFW, TID and TED (r ≥ 0.85, equivalent to r^2^ ≥ 0.72). We note that the most reproducible indices of visceral fat all relate to body cavity CSA. By assuming body cavity CSA takes the form of an ellipse, we estimated this as body cavity CSA = π x (SD – 2SFW) × TID for our validation sample.

**Table 3:** Visceral adipose tissue area (VAT area) linear model estimates and correlational indices

	**Model**	**Reported *R***^**2**^	**TwinsUK *R***^**2**^
**A**			
Snijder *et al.* (2002) [11]	DXA trunk fat + sagittal depth	0.74	0.80
	DXA trunk fat + abdominal circumference	0.71	0.78
Treuth *et al.* (1995) [15]	Sagittal depth + age + waist circumference +% DXA trunk fat	0.81	0.79
Hill *et al.* (2007) [10]	DXA + skin fold	0.68	0.65
**B**	**Index**	**Reported *r***	**TwinsUK**
Bertin *et al.* (2002) [8]			***r***
	Abdominal fat mass (kg)	0.57	0.79
	Thigh fat mass (kg)	0.06*	-
	Abdominal fat mass/thigh fat mass	0.75	-
	Abdominal fat mass/SFW	0.83	0.58
	TED (cm)	0.54	0.61
	TID (cm)	0.9	0.61
	SFW (cm)	−0.23*	0.28
	(SD)(TID)	0.89	0.87
	(SD)(TID)/height	0.91	0.86
	(SD)(TID)/BMI	0.66	0.49
	(SD-SFW)	0.86	0.89
	(SD-SFW)(TID)	0.92	0.79
	(SD-SFW)(TID)/height	0.94	0.87

Previously reported models in the literature were applied to the TwinsUK validation sample of CT-measured VAT area (n = 54) and the coefficient of determination (*R*^2^) is presented for each study as an indication of the proportion of VAT variance explained by the model. (**A**). The Pearson product–moment correlation coefficients between CT-measured VAT area and adiposity indices described in Bertin *et al.* (2002) were also calculated for the TwinsUK validation sample (**B**). Note that the reported linear models all replicate using TwinsUK and that highest correlational indices (r > 0.85) for VAT area were all anthropometric measures that relate to size of the body cavity area. Skin fold was estimated using the formula SFW = (TED – TID)/2 applied to DXA data, as calliper skin fold measure was not taken for the TwinsUK study. Asterisks in table B indicate the reported correlation coefficient does not differ significantly from zero (at threshold α = 0.05). Abbreviations: *DXA*, dual-energy X-ray absorptiometry, *SD*, sagittal depth, *SFW*, subcutaneous fat width, *TED*, transverse external diameter, *TID*, transverse internal diameter.

In modelling CT-measured VAT area, the best fit and most interpretable model included a combination of measures for DXA abdominal fat, body cavity cross sectional area (estimated using the ellipse formula above) and waist circumference (Table 4, Model 0), which together explained 91% of the variance in CT-measured VAT area (*R*^2^ = 0.91). However, since sagittal depth was not available for our study sample for which we wished to estimate VAT, we also assessed a model including only DXA total abdominal fat, tape-measured waist circumference and age. For this (Table 4, Model 1) we obtained a model with an *R*^2^ of 0.83 with the following linear regression equation: VAT area = 10.1(DXA abdominal fat mass) + 40.8(waist circumference) + 1.4(age) using standardised explanatory variables and no intercept term. For this estimate, a Bland-Altman analysis showed no evidence of heteroscedascity across the full range of CT-measured VAT area, with only 2 out of 54 (3.7%) values with a difference outside the 95 limits of agreement (the mean difference ± twice the standard deviation of the difference between the two measures).

**Table 4:** Linear regression models for CT visceral adipose fat (VAT) area using the validation sample (n = 54)

	**VAT model**	**Measure**	**β**	**SE**	**t**	***p *** **value**	**95% CI**		**Model**
							**Lower**	**Upper**	***R***^**2**^
**A**	**Model 0:**								0.91
	Combination of DXA & anthropometric measures	DXA abdominal fat	20.1	3.4	5.9	2 × 10^-9^	13.2	27.0	
		BC CSA	32.4	4.5	7.2	4 × 10^-13^	23.2	41.6	
		WC	11.1	5.6	2.0	2 × 10^-2^	-0.3	22.4	
**B**	**Model 1:**								0.83
	Combination of DXA & anthropometric measures	DXA abdominal fat	10.1	4.8	2.1	0.04	0.31	19.9	
		WC	40.8	5.7	7.2	3 × 10^-13^	29.2	52.3	
		Age	1.4	0.5	2.6	0.01	0.3	2.4	
**C**	**Model 2:**								0.86
	Anthropometric measures only	BC CSA	25.5	5.6	4.6	2 × 10^-6^	14.1	36.8	
		WC	30.5	5.5	5.6	1 × 10^-8^	19.4	41.6	

Model 0: combination of DXA and anthropometric measures guided by previously published models presented in Tables 3A (**A**); Model 1: combination of DXA and anthropometric measures restricted to DXA total abdominal fat, WC and age that were also available for the study sample (**B**); Model 2: using anthropometric measures only (**C**). BC CSA was estimated using BC = (π × (SD–2SFW) × TID) from the CT images at intervertebral disc L4:L5 as described in Methods. Note that for Model 2, using these explanatory variables instead of BC CSA, yields equally good or better prediction of VAT area (*R*^2^ = 0.89), but the model is less interpretable with a negative beta coefficient for SFW. Abbreviations: *BC*, body cavity, *CSA*, cross sectional area, *SD*, sagittal depth, *SFW*, subcutaneous fat width, *TED*, transverse external diameter, *TID*, transverse internal diameter.

In relation to efforts attempting to estimate visceral fat using only anthropometric measures [8,33], we also obtained a highly explanatory model (*R*^2^ = 0.86) with a linear equation using only two CT measures of body cavity CSA (estimated as an ellipse) and waist circumference (Table 4, Model 2)*.* This figure rose to *R*^2^ = 0.89 using body cavity components SD, SFW and WC as explanatory variables for CT VAT area (data not shown). Body cavity CSA results are presented, as this model is more interpretable, while the model including SD, SFW and WC yields a negative beta coefficient for SFW due to co-linearity. Again, these simple anthropometric models could not be used for the study sample however, as sagittal depth was not recorded along with DXA scan for these subjects.

In addition to these validation models, we indirectly assessed the validity of our study sample estimates of visceral fat by making two observations:

1. Realised estimates of VAT area (VAT_Model1_ and VAT_Model2_) for the validation sample were equally or more strongly correlated with VAT_CT_ (*r =* 0.89 and *r =* 0.93) than DXA total abdominal fat (*r* = 0.88 and *r* = 0.70, respectively);

2. Bivariate variance component analysis between VAT_Model1_ and DXA total abdominal fat provided strong statistical evidence (Δχ^2^_1_ = 43.7, p = 4x10^-11^) for a specific heritable component that was unique to VAT_Model1_ and not shared with DXA total abdominal fat (data not shown).

### Heritability (study sample)

The data provided a narrow sense heritability estimate of 0.58 (95% CI: 0.51-0.66) and a familial environmental effect of 0.24 (95% CI: 0.17-0.30) for VAT area (Table 5), with the best fit to the data being the ACE model, including additive polygenic (A), common familial (C) and unique environmental (E) components.

**Table 5:** **Visceral adipose tissue (VAT) area estimate of heritability (*h***^**2**^**) and model fit statistics (n = 3457)**

**Model**	**−2 LL**	**df**	**AIC**	**Δdf**	**Δχ**^**2**^	***p *** **value**		***h***^**2**^	**95% CI**
ACE	51399.9	4737	41925.9	-	-	-	A	0.58	(0.51-0.66)
							C	0.24	(0.17-0.30)
							E	0.18	(0.16-0.20)
AE	51438.7	4738	41962.7	1	39	4.70x10^-10^	A	0.83	(0.81-0.84)
							C	-	-
							E	0.18	(0.16-0.19)
CE	51608.4	4738	42132.4	1	209	2.92x10^-47^	A	-	-
							C	0.62	(0.59-0.64)
							E	0.38	(0.36-0.41)

Full (ACE) and nested (AE and CE) model estimates are presented. Nested sub-models test the hypothesis that the estimated additive polygenic genetic variance component (model CE) and the shared familial environmental component (model AE) do not contribute to the observed phenotypic variance. The full ACE model is the best-fit model, since the more parsimonious sub-models do not fit the data as well (p < < 0.05). The model with the lowest AIC fit statistic also indicates best model fit. Abbreviations: -2 LL: minus twice the log-likelihood; AIC: Akaike’s Information Criterion; Δ*χ*^2^: likelihood ratio chi square statistic; A – additive polygenic variance component, C – common familial environment, E – unique environmental variance (and measurement error) specific to the individual.

### Visceral fat as a risk factor of morbidity (study sample)

For the study sample we estimated visceral fat as a linear function of DXA total abdominal fat, waist circumference and age (Model 1, Table 4). Although a model including DXA total abdominal fat, waist circumference and estimated body cavity area was a better fit (Model 0, Table 4), we used Model 1 as we did not have a measure of sagittal depth for the study sample subjects required to estimate body cavity area from the DXA images.

The correlations between study sample explanatory variables are presented in Additional file 1: Table S1. As a function of these variables, VAT area is most strongly correlated with waist circumference and DXA total abdominal fat. While the univariate odd ratios for VAT area, DXA abdominal fat and BMI were all significantly associated with each morbidity (Tables 6, 7, 8 and 9), VAT area was most consistently and strongly associated with four morbidity traits - T2D, HT, cIMT and ALT. Residual analyses for these four morbidities were consistent with visceral fat entirely mediating the association with other measures of adiposity (BMI and total abdominal fat), but this was not true for the liver function tests ALK, BIL and GGT (details provided in supplementary Tables online).

**Table 6:** Type 2 diabetes (T2D) and adiposity

**Type 2 diabetes**		**OR**	**SE**	**z**	***p *** **value**	**95% CI**		**Model**
						**Lower**	**Upper**	**pseudo-*R***^**2**^
**A**								
	VAT	2.17	0.18	9.5	<2 × 10^-16^	1.85	2.54	0.07
	DXA abdominal fat	1.86	0.13	8.6	<2 × 10^-16^	1.61	2.14	0.05
	BMI	1.66	0.12	7.2	2 × 10^-13^	1.45	1.91	0.04
	Age	1.05	0.01	4.3	8 × 10^-6^	1.03	1.07	0.02
**B**								
	VAT	2.08	0.18	8.5	<2 × 10^-16^	1.76	2.47	0.08
	Age	1.02	0.01	2.0	0.05	1.00	1.05	

The study sample prevalence (females > = 40 years) estimate for T2D = 0.05. Logistic regressions (n = 2964) presenting unadjusted odds ratios (OR) (A) and best-fit multiple regression model with adjusted OR for visceral adipose fat (VAT) area and age (B). For evidence of the presented best-fit model and an analysis of residuals to account for co-linearity between adiposity variables, see Additional file 1: Tables S2 and S3, respectively. Explanatory variables VAT, DXA and BMI are all standardised, implying a change in odds ratio per unit SD change. For logistic regression, the pseudo-*R*^2^ model-fit statistic is analogous (but not directly comparable) to the ordinary least squares regression *R*^2^ statistic, known as the coefficient of determination. While *R*^2^ can be interpreted as the proportion of variance explained by the model, pseudo-*R*^2^ is loosely interpreted as the proportion of variation in risk liability explained by the model (StatCorp, Texas). Abbreviation: CI - confidence interval.

**Table 7:** Hypertension and adiposity

**Hypertension**		**OR**	**SE**	**z**	***p *** **value**	**95% CI**		**Model**
						**Lower**	**Upper**	**pseudo-*R***^**2**^
**A**								
	VAT	2.08	0.16	9.5	<2 × 10^-16^	1.79	2.42	0.08
	DXA abdominal fat	1.77	0.14	7.4	6 × 10^-14^	1.53	2.07	0.05
	BMI	1.77	0.13	7.7	6 × 10^-15^	1.53	2.05	0.06
	Age	1.07	0.01	6.4	6 × 10^-11^	1.05	1.09	0.05
**B**								
	VAT	1.90	0.17	7.4	9 × 10^-14^	1.60	2.25	0.10
	Age	1.04	0.01	4.0	4 × 10^-5^	1.02	1.07	

The study sample prevalence estimate (females > = 40 years) for hypertension = 0.08. Logistic regressions (n = 2032) showing unadjusted ORs (A) and best fit multiple regression model including visceral adipose fat (VAT) area and age (B). For evidence of the presented best-fit model and an analysis of residuals to account for co-linearity between adiposity variables, see Additional file 1: Tables S4 and S5, respectively. Explanatory variables VAT, DXA and BMI are all standardised, implying a change in odds ratio per unit SD change. Year of visit was categorised as quintiles and included in all HT analyses as a categorical confounding variable. See Table 6 legend for an explanation of pseudo-*R*^2^. Abbreviation: *CI*, confidence interval.

**Table 8:** Sub-clinical atherosclerosis and adiposity

**Carotid intima-media thickness**		**HR**	**SE**	**z**	***p *** **value**	**95% CI**		**Model fit (Wald)**		
						**Lower**	**Upper**	**χ**^**2**^	**df**	***p *** **value**
**A**										
	VAT	1.50	0.09	6.6	2 × 10^-11^	1.33	1.69	43.8	1	4 × 10^-11^
	DXA abdominal fat	1.29	0.07	4.5	4 × 10^-6^	1.16	1.45	20.0	1	8 × 10^-6^
	BMI	1.39	0.08	5.5	2 × 10^-8^	1.23	1.55	30.5	1	3 × 10^-8^
	Age	1.08	0.01	6.3	1 × 10^-10^	1.05	1.10	40.0	1	3 × 10^-10^
**B**										
	VAT	1.36	0.10	4.4	5 × 10^-6^	1.19	1.56	59.6	2	1 × 10^-13^
	Age	1.06	0.01	5.0	3 × 10^-7^	1.04	1.09			

Cox proportional hazards regression (*n* = 801) showing unadjusted ORs (**A**) and best-fit model including visceral adipose fat (VAT) area and age (**B**). For evidence of the presented best-fit model and an analysis of residuals to account for co-linearity between adiposity variables, see Additional file 1: Tables S6 and S7, respectively. The study sample prevalence estimate (females > = 40 years) for sub-clinical atherosclerosis at follow-up was 0.27 (average time from baseline to follow up was 9.95 years, range 5–16 years). Explanatory variables VAT, DXA and BMI are all standardised, implying a change in hazard ratio per unit SD change. For Cox proportional hazards, the Wald model-fit statistic is presented to indicate the best model fit (StatCorp, Texas) that predicts onset of sub-clinical atherosclerosis (carotid intima-media thickness, cIMT). Abbreviation: CI - confidence interval.

**Table 9:** Liver function tests (LFTs) and adiposity

**Liver function tests**		**OR**	**SE**	**z**	***p *** **value**	**95% CI**		**Model pseudo-*R***^**2**^
						**Lower**	**Upper**	
ALT (0.22)								0.09
	VAT	1.75	0.09	10.9	<2 × 10^-16^	1.58	1.93	
	Age	1.02	0.01	2.9	0.004	1.01	1.03	
ALK (0.27)								0.09
	VAT	1.28	0.14	2.4	0.02	1.05	1.58	
	DXA abdominal fat	1.26	0.13	2.3	0.02	1.03	1.54	
	Age	1.06	0.01	9.8	<2 × 10^-16^	1.05	1.07	
BIL* (0.02)								0.054
	VAT	0.62	0.10	−3.0	0.003	0.46	0.85	
BIL* (0.02)								0.049
	DXA abdominal fat	0.67	0.10	−2.7	0.01	0.50	0.90	
GGT (0.20)								0.06
	VAT	1.25	0.14	1.9	0.05	1.00	1.56	
	DXA abdominal fat	1.36	0.15	2.8	0.01	1.10	1.70	
	Age	1.02	0.01	2.7	0.007	1.00	1.03	

Best-fit multiple regression models for LFTs are presented for the logistic regression models (n = 3014) including potential explanatory variables visceral adipose tissue (VAT) area, DXA total abdominal fat, body mass index (BMI) and age. Prevalence for upper limit of normal threshold for each assay is indicated in brackets (see Methods). Explanatory variables VAT, DXA and BMI are all standardised, implying a change in odds ratio per unit SD change. Year of visit was categorised as quintiles and included in all LFT analyses as a categorical confounding variable. *Note that two multiple regression models are presented for BIL, since both DXA and VAT area predict BIL equally well. Including both measures in this model provides uninterpretable ORs due to co-linearity between the variables (see Additional file 1: Table S1). For evidence of the presented best-fit model and analysis of residuals to account for co-linearity between adiposity variables for the four LFTs, see Additional file 1: Tables S8-S15. Abbreviations: *ALT*, alanine transaminase, *ALK*, alkaline phosphatase, *BIL*, bilirubin, *CI*, confidence interval, *GGT*, gamma-glutamyl transpeptidase.

For type 2 diabetes, while the univariate ORs for the 3 adiposity measures were all associated with T2D (Table 6A), visceral fat and age provided the best-fit multiple regression model (pseudo-*R*^2^ = 0.08, Table 6B and LRT, Additional file 1: Table S2), with an adjusted OR of 2.08 (95% CI 1.76 – 2.47) per standard deviation increment in VAT area including age. Removing VAT area from the full model resulted in a significant decline in model fit (LRT χ^2^_1_ = 14.4, p = 1E-04), while the removal of DXA total abdominal fat and BMI, either individually or together (χ^2^_2_ = 1.9, p = 0.39) did not reduce the model fit (Additional file 1: Table S2).

Hypertension was equally strongly associated with VAT area, DXA abdominal fat and BMI for univariate analyses (Table 7A), but visceral fat and age provided the best-fit multiple regression model (pseudo-*R*^2^ = 0.10, Table 7B and LRT, Additional file 1: Table S4), with an adjusted OR of 1.90 (95% CI 1.60 – 2.25) per standard deviation increment in VAT area including age. Removing VAT area from the full model resulted in a nominal decline in model fit (LRT χ^2^_1_ = 7.1, p = 0.01), whilst the removal of DXA total abdominal fat and BMI, either individually or together (χ^2^_2_ = 1.99, p = 0.37) did not reduce the model fit (Additional file 1: Table S4).

The prospective analysis of subclinical atherosclerosis had a median follow up time of 9.7 years, during which a total of 221 (27%) individuals were classified as sub-clinically atherosclerotic. Univariate Cox proportional hazard models showed all three measures of adiposity and age at baseline to be associated with incident cIMT (Table 8A), with VAT area (χ^2^_1_ = 43.8) and age (χ^2^_1_ = 40) providing the best-fit parsimonious model (Table 8B). DXA and BMI could be dropped from the full model with no nominal (χ^2^_2_ = 5.7, p = 0.06) deterioration in model fit (Additional file 1: Table S6).

All LFT protein serum levels were positively associated with measures of adiposity (Table 9), except for bilirubin, which was negatively associated. VAT area remained associated with alanine transaminase (ALT) when conditioned upon DXA total abdominal fat and BMI, while VAT area and DXA were still associated with ALK, BIL and GGT conditional upon BMI (Table 9 and related Additional file 1: Tables S8-S15). Removing VAT area from the full model for ALT resulted in a significant decline in model fit (LRT χ^2^_1_ = 19.6, p = 1E-05), while the removal of DXA total abdominal fat and BMI, either individually or together (χ^2^_2_ = 1.56, p = 0.46) did not (Additional file 1: Table S8).

The estimated variance inflation factor (VIF) between VAT area and DXA abdominal fat, BMI and age was 8.84. Analyses repeated using subgroups of data and analyses using underlying quantitative traits for HT, ALT, ALK, BIL and GGT all provided qualitatively the same association results (data not shown).

## Discussion

This study shows that DXA and anthropometric measures can be used to derive reliable estimates of visceral fat and provides evidence that visceral fat - independent of BMI and total abdominal body fat - is a risk factor for type 2 diabetes, hypertension, subclinical atherosclerosis and ALT liver function. Validation sample explanatory models were able to explain >80% of the variance in CT-measured visceral fat. A combination of DXA and anthropometric measures (*R*^2^ = 0.91) or models including only anthropometric measures (*R*^2^ = 0.86 – 0.89), both provided equally good estimates of visceral fat. We obtain a heritability of 58% for visceral fat, which is consistent with familial studies that report within the region of 48-57% [34-37] and using a twin design, confirms for the first time that the observed familial component is also partly due to shared familial environment (24%).

Our results confirm that visceral fat is the single most important measure of adiposity for risk of type 2 diabetes. In large DXA resources, such as the one used by Leslie *et al.* (2010) [38], it would be interesting to ascertain whether the risk of developing diabetes is better predicted if a DXA-based estimate of visceral fat was used rather than using DXA total abdominal fat.

Visceral fat mediates all associations between hypertension and adiposity variables for these cross sectional data. Similarly, in a longitudinal study, Hayashi *et al.* (2004) [39] showed that only visceral fat is significantly associated with hypertension, when visceral fat is adjusted for other adiposity measures (e.g. BMI, waist circumference or abdominal subcutaneous fat). Visceral and subcutaneous adiposity both contribute to the prevalence of hypertension, but when adjusted for BMI or waist circumference, subcutaneous fat is no longer associated with hypertension [37]. This further illustrates that it is the distribution between these two fat depots that appears to determine the risk of morbidity.

The analysis of incident subclinical atherosclerosis is consistent with cross-sectional studies that show association between atherosclerosis, total abdominal fat [40] and visceral fat [41,42]. Here we provide additional evidence that total abdominal and visceral fat accumulation precedes atherosclerosis.

The LFT protein results suggest that liver function deteriorates with increasing visceral adiposity with VAT area positively associated with ALT, ALK and GGT. By contrast, the inverse relationship between visceral fat and bilirubin is consistent with the inverse association between bilirubin, insulin resistance [43] and GGT activity [44]. In particular, of the four tests, the linear association between ALT and adiposity is specifically mediated via visceral fat, while for ALK, BIL and GGT there is only evidence to suggest this is the case for abdominal fat *per se*. The visceral adipose depot is thought to be the major source of elevated fatty acid delivered to the liver via the portal vein and it is possible that visceral fat acts as a marker of hepatic fat content [45]. Hepatic fat accumulation is known to impair insulin signalling in hepatocytes [46,47] and “pathway selective insulin resistance” [48] maybe pivotal in the transition from normal to impaired fasting glucose states. It has been hypothesised that non-alcoholic fatty liver (NALFD) may play a mediatory role in the pathology of CVD [49].

The relative strengths and limitations of this study were: 1) Adiposity inferences are restricted to middle-aged Caucasian women with a BMI ≤ 35. The VAT area estimates for the study sample included the 4% of females with a BMI > 35, which was outside the validation sample data range. Although these estimates strictly represent an invalid data extrapolation, their exclusion did not qualitatively alter the results for the heritability or morbidity association (data not shown) and so these individuals were retained. 2) We have shown that sagittal depth is an extremely useful yet simple anthropometric measurement to estimate the body cavity area. When used in combination with the subcutaneous fat width, this measure contributes to a very good estimation of visceral fat (*R*^2^ approximately 90%). Where DXA is not available, sagittal depth, waist circumference and subcutaneous fat width could therefore be measured to quickly and inexpensively estimate a person’s visceral fat content with just a tape measure and skin callipers. Not having sagittal depth for the study sample limited our ability to estimate visceral fat more accurately. This is indicated by the best anthropometric validation sample estimate (Model 2) predicting VAT area as well or better than a combination of DXA total abdominal fat, waist circumference and age (Model 1); 3) Arising from the second limitation, although our prediction of visceral fat was still valid [32], co-linearity between model explanatory variables – in particular VAT area and DXA total abdominal fat – meant that adjusted Odds Ratios for risk factors were not interpretable. Instead, conditional independence structures were assessed using residuals analysis. This allowed us to eliminate the possibility of causal relationships between adiposity variables and morbidity, except those mediated by visceral fat; 4) The twin-based heritability estimate presented here is likely to be an under-estimate, since random measurement error is included in the denominator of the heritability ratio estimate and the study sample estimate of visceral fat is known to account for approximately only 83% of CT-measured VAT area; 5) The study design for incident subclinical atherosclerosis was not strictly prospective, since no baseline measure of cIMT was taken to facilitate the exclusion of potential baseline cases. Where undetected baseline cases exist, the study design is cross sectional; 6) Inclusion criteria for the validation sample were that study subjects were female, over 40 and had both DXA and CT scans within 2 years of one another. Although not measured the same day, time between visits was included in analyses as a confounding variable and Bland-Altman plots revealed no bias in the relationship between predicted and CT-measured visceral fat values. More importantly, no significant weight gain was observed between the collection of CT and DXA measurements and small fluctuations in weight were not correlated with the time lapse between scan dates.

This study shows that, where a direct measure of visceral fat is unavailable, using an indirect estimate of visceral fat (even without a measure of subcutaneous fat) is more predictive of morbidity than total abdominal fat. Our study suggests that both DXA body fat composition and anthropometric measures of body cavity volume alone can provide reliable estimates of abdominal visceral fat.

In conclusion, we demonstrate that DXA-based measures of visceral fat can be both reliable and valid and are a cheaper and less intrusive alternative to other methods such as MRI and CT to estimate adiposity. Visceral fat is not only strongly associated with morbidity, but also appears to mediate the association between morbidity and other measures of adiposity for type 2 diabetes, hypertension and elevated alanine transaminase serum levels. This is consistent with hypotheses that suggest excess visceral fat accumulation is causally related to cardiovascular and metabolic disease.

## Abbreviations

ALK: Alkaline phosphatase; ALT: Alanine transaminase; BIL: Total bilirubin; cIMT: Carotid intima-media thickness (subclinical atherosclerosis); CT: Computed tomography; CSA: Cross sectional area; DXA: Dual-energy X-ray absorptiometry; GGT: Gamma-glutamyl transpeptidase; HT: Hypertension; SFW: Subcutaneous fat width; TID: Transverse internal diameter; TED: Transverse external diameter; T2D: Type 2 diabetes; VAT: Visceral adipose tissue.

## Competing interests

The author declare that they have no competing interest.

## Authors’ contributions

KD - design of the experiment, collection of data, analysis of data, writing of the manuscript; MC – collection of data; TDS, PC – collection of data, comments on the manuscript; WA, MF – analysis of data, comments on the manuscript; TA: design of the experiment, collection of data, analysis of data, writing of the manuscript. All authors read and approved the final manuscript.

## Pre-publication history

The pre-publication history for this paper can be accessed here:

http://www.biomedcentral.com/1471-2261/13/25/prepub

## Supplementary Material

Additional file 1: Table S1 Study sample (n = 3457) correlation and colinearity between measures of adiposity. **Table S2**. Type 2 diabetes (T2D) multiple regression analyses. **Table S3**. Residuals analysis for type 2 diabetes (T2D). **Table S4**. Hypertension (HT) multiple regression analyses. **Table S5**. Residuals analysis for hypertension (HT). **Table S6**. Carotid intima-media thickness (cIMT) Cox multiple regression analyses. **Table S7**. Residuals analysis for carotid intima-media thickness (cIMT). **Table S8**. Multiple regression analyses for alanine transaminase (ALT). **Table S9**. Residuals analysis for the quantitative trait (QT)/assay for alanine transaminase (ALT). **Table S10**. Multiple regression analyses for alkaline phosphatase (ALK). **Table S11**. Residuals analysis for the quantitative trait (QT)/assay alkaline phosphate (ALK). **Table S12**. Multiple regression analyses for bilirubin (BIL). **Table S13**. Residuals analysis for the quantitative trait (QT)/assay for bilirubin (BIL). **Table S14**. Multiple regression analyses for gamma-glutamyl transpeptidase (GGT). **Table S15**. Residuals analysis for the quantitative trait (QT)/assay gamma-glutamyl transpeptidase (GGT). Click here for file
